# Supplementary material for: Social buffering in rats reduces fear by oxytocin triggering sustained changes in central amygdala neuronal activity
Source: Nat Commun. 2024 Mar 7;15:2081. doi: 10.1038/s41467-024-45626-z (PMC10920863; doi:10.1038/s41467-024-45626-z)
Supplement: Supplementary file 1 — Supplementary Information [file 41467_2024_45626_MOESM1_ESM.pdf]

Supplementary information

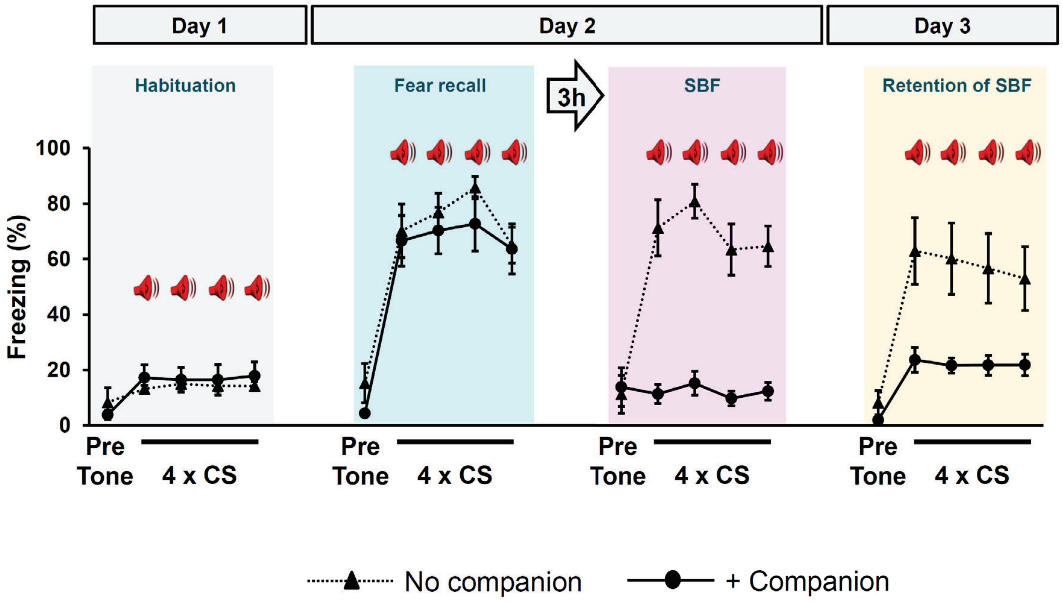

Supplementary Figure 1: Effects of SBF and retention of SBF are immediate, starting from the first representation of the CS.

Representation of same results as in Fig. 1b, but with the values of freezing responses to the individual 4 consecutive exposures to CS1 that receive SBF on Day 2 and that are separately averaged per presentation. The presence of the companion on Day 2 (SBF) does not gradually decrease freezing to the CS, but leads to an immediate decrease in freezing levels (i.e. with the start of the first CS exposure). On Day 3, the maintenance of this decrease is also immediate (occurring upon exposure to the first CS) and maintained at equal low level throughout subsequent exposures. Mean values ±SEM are shown.

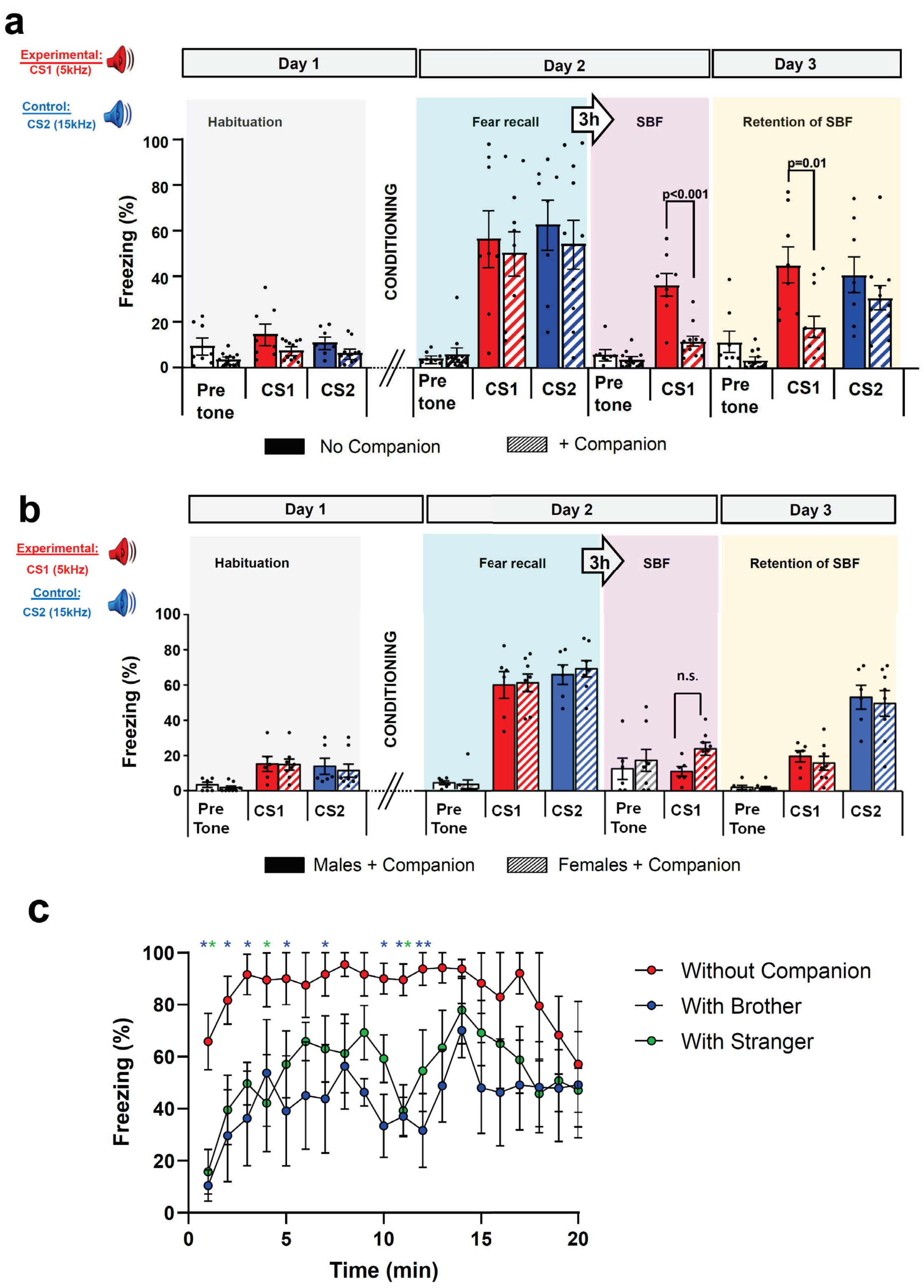

Supplementary Figure 2: Immediate and long-lasting effects of SBF in animals under different housing conditions or of different sex.

(a) In group-housed animals the presence of the companion acutely and long-lastingly reduces freezing similar to all other previously obtained results. Repeated measures two-way ANOVA; $F(3,34)=28.87$, $p<0.001$ (SBF) and $F(5.51)=9.373$, $p<0.001$. Bonferroni-corrected significance in the figures. n=8 no companion, n=11 + companion (**b**) Animals of different sex exhibit acutely and long-lastingly reduced freezing levels to similar extent in the presence of a companion of the same sex (males: filled bars; females: striped bars). For both (a) and (b), rats were exposed (as described in protocol in Figure 1a) during habituation on Day 1 four times to two different auditory conditioned stimuli (CS1: 5 kHz, red; CS2: 15 kHz, blue) and subsequently fear conditioned by pairing each CS with an electric foot shock of 0.5 mA. On Day 2 memory of fear was assessed by re-exposure to each CS (fear recall). 3 hours later, rats were habituated to a polyester ball (n=6) or a companion rat in the adjacent compartment (n=6) for 10 min and subsequently re-exposed to the CS2 (SBF). On Day 3, both CSs were presented again in the absence of a companion (Retention of SBF). Freezing was diminished only for the CS (CS2) that was paired with social buffering. Conventions as in Fig. 1. Two-way ANOVA with repeated measures (pre-tone, CS1, CS2) and group ("+ No companion, + Companion"), for each session (Habituation, Fear recall, SBF, Retention of SBF). Individual and mean values ± SEM are shown. **(c)** SBF works equally well in the presence of a familiar or unfamiliar conspecific in a contextual fear conditioning paradigm. Two-way ANOVA followed by Bonferroni correction for multiple comparisons; *, $p<0.05$; **, $p<0.01$; blue asterisks, Brother vs Without Companion; green asterisks, Stranger vs Without Companion. n=4 for all groups.

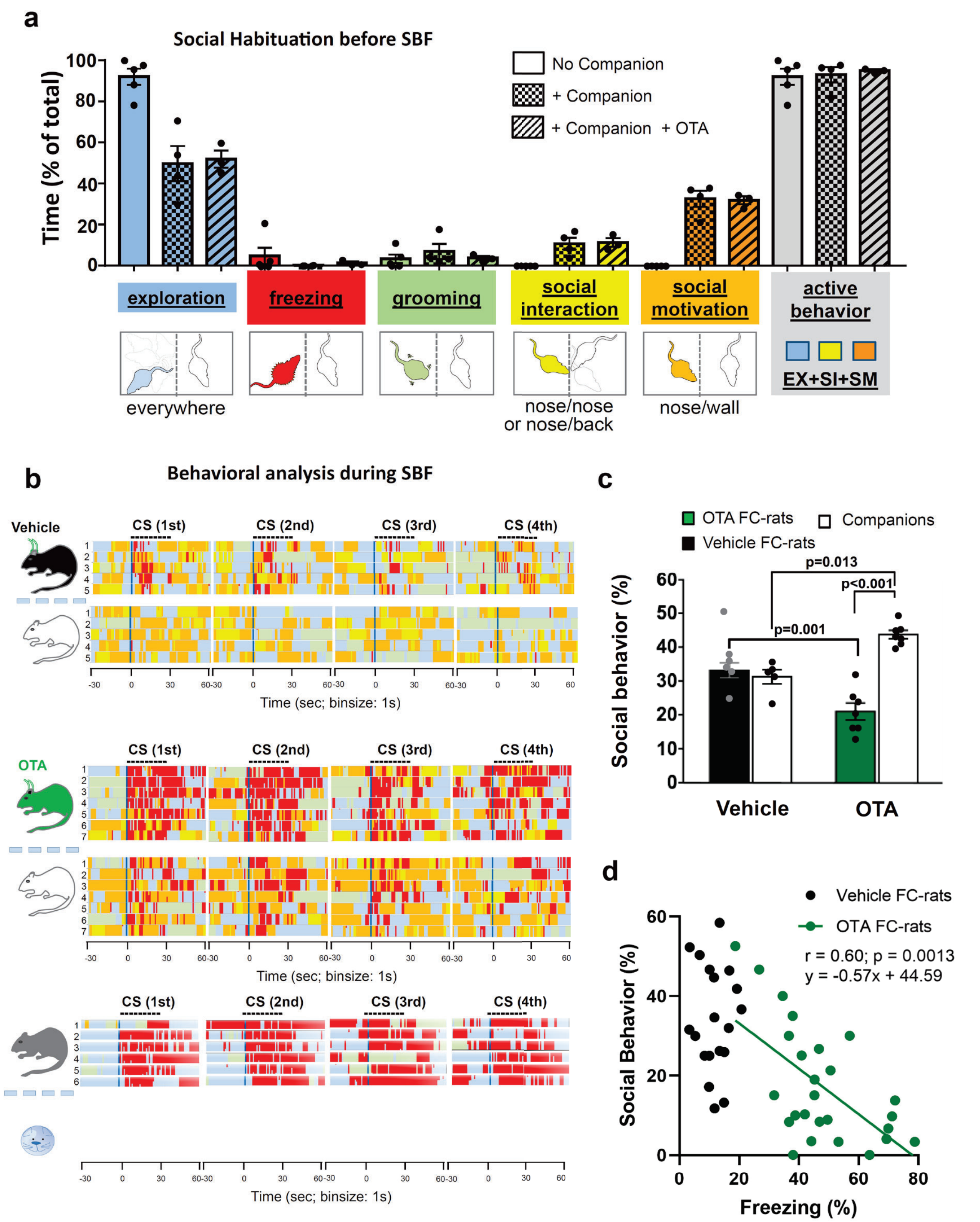

Supplementary Figure 3 | Analysis of behavioral interactions between fear-conditioned and companion rats during different experimental paradigms.

(**a**) Bar chart of behavioral activity of demonstrator rat in absence or presence of companion rat before recall of the CS on Day 2 with and without injection of OTA. For these analyses, we assessed the behavior every second using the following categories: "exploration" ("EX"),

"freezing", "grooming" (licking of entire body), "social interaction" (nose to nose or nose to body through the plexiglass wall, "SI"), "social motivation" (close to the plexiglass wall, "SM"). Active behavior was defined as the total time spent in exploration, social interaction and social motivation. Individual and mean values ± SEM are shown (n=5-7) **(b)** Ethograms of color-coded behaviors before, during and after 4 CS presentations to FC-rats and their companion with each line at similar vertical position in upper and lower blocks representing behavior of resp. an individual FC-rat and its corresponding companion. First series: vehicle injected in CeA of FC-rat (black, n=5), second series: OTA injected in FC-rat (green, n=7), third series: FC-rat exposed to polystyrene ball (grey, n=6). For these analyses, we assessed the behavior every second (bin-size of ethograms=1 sec) **(c)** Bar chart of average percentage of time exhibiting social behavior (="social interaction" + "social motivation") by FC-rats injected with vehicle (black) or OTA (green) and their corresponding companions (white). One-way ANOVA, $F(3,21)=17.41$, $p<0.001$; p values following Bonferroni corrections for multiple comparisons in the graph. **(d)** Scatter plot of social behavior levels (as defined in (c) as a function of freezing levels in FC-rats injected with vehicle (black) or OTA (green)).

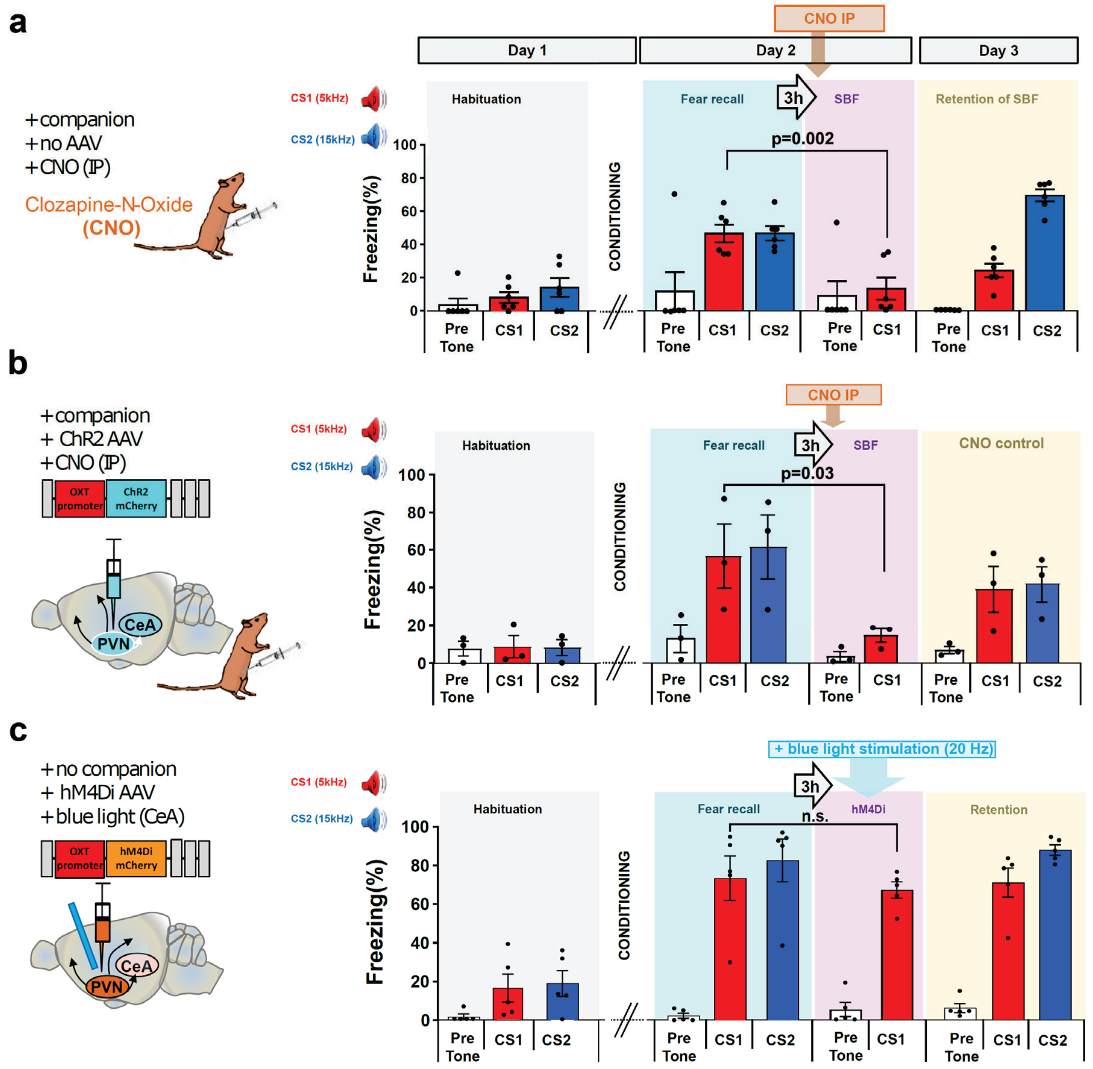

Supplementary Figure 4: Chemo,- and optogenetic effects are specific to viral expression.

(a) IP CNO does not disrupt SBF in the absence of DREADD expression. CNO, given IP 30 min before SBF does not affect decreased freezing levels during "SBF" and "retention of SBF" (CS1 fear recall vs CS1 SBF, One-tailed Student's t-test, t=3.876, df=10, p value in figure). (b) IP CNO does not disrupt SBF when PVN neurons express ChR2 (instead of hM4Di) (CS1 fear recall vs CS1 SBF, One-tailed Student's t-test, t=3.876, df=10, p value in the figure). (c) Blue light during exposure to the CS does not significantly decrease freezing when oxytocinergic PVN neurons express hM4Di. (CS1 fear recall vs CS1 SBF, One-sided Student's t-test, t=0.12, df=8, p=0.454 (n.s.)) For all experiments: n=3-6 animals (as

indicated by the dots). Insets on the left shows virus injections sites and concomitant treatments. See also[1].

.

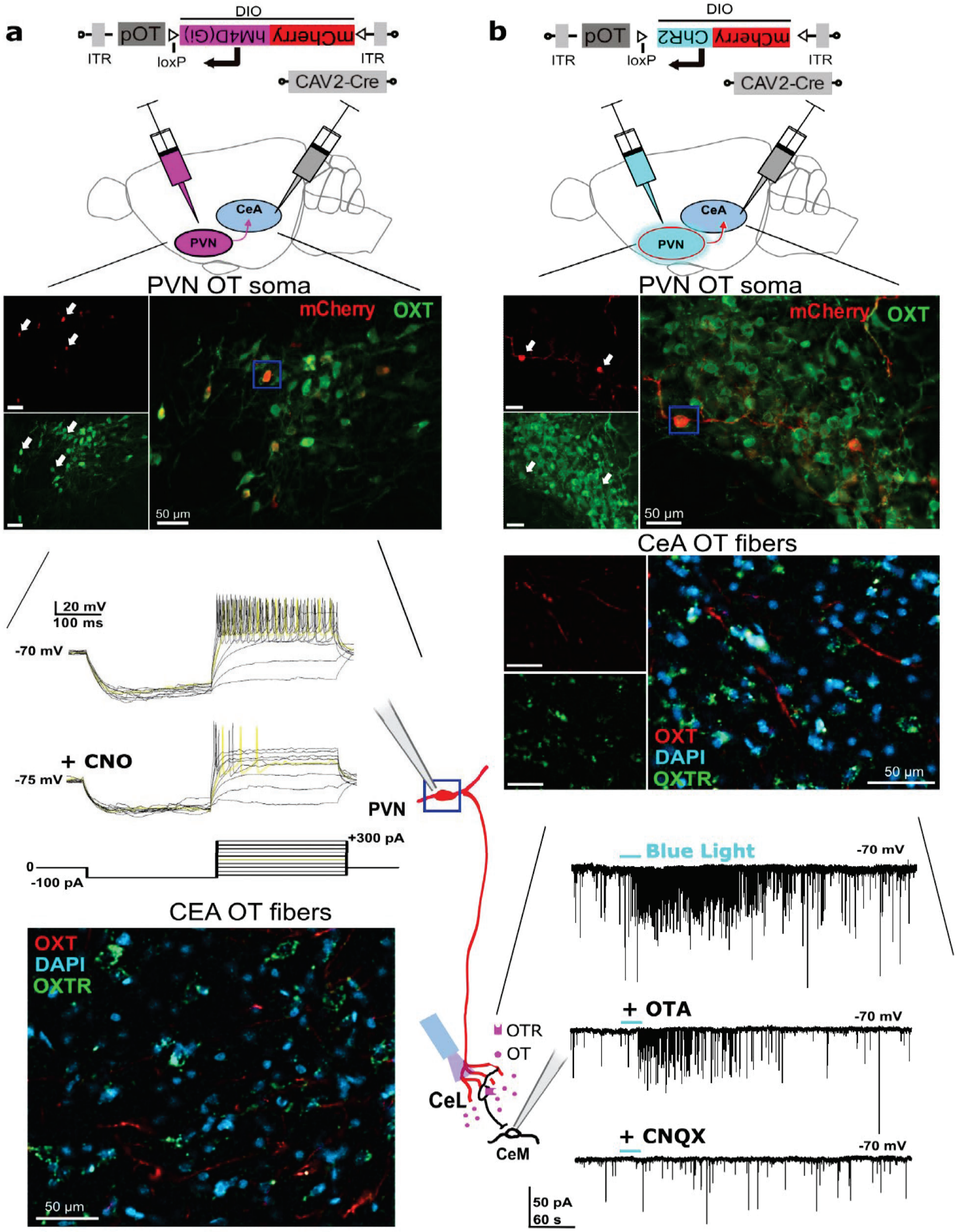

Supplementary Figure 5: *In vitro* and *in vivo* electrophysiological recordings in PVN and CeA neurons demonstrating their opto,- and chemogenetic modulation

(a) Combined genetic-retrograde labeling by virus injection of AAV-expressing double-floxed DIO mCherry-hM4Di under OT promoter (pOT) in PVN and CAV2-expressing CRE in CeA ensuring hM4Di expression specifically in PVN OT neurons projecting to CeA. Inset below: Antibody labeling of PVN neurons expressing AAV virus (mCherry, red; Oxytocin, green).

Below: *in vitro* electrophysiological recordings of PVN neuron expressing mCherry-fluorescent hM4Di, demonstrating decreased activation after CNO perfusion and consequently decreased OT release. Inset: staining of OTergic fibers in the CeA (mCherry, red, by viral infection) and oxytocin receptors (by RNAscope, green) **(b)** Combined genetic-retrograde labeling by virus injection of AAV-expressing double-floxed DIO mCherry-ChR2 under OT promoter (pOT) in PVN and CAV2-expressing CRE in CeA ensuring ChR2 expression specifically in PVN OT neurons projecting to CeA. Inset below: Antibody labeling of PVN neurons expressing AAV virus (mCherry, red); Oxytocin, green). Below: staining of OTergic fibers in the CeA (mCherry, red, by viral infection) and oxytocin receptors (by RNAscope, green). Traces below show functional expression in an *in vitro* electrophysiological slice recording of a neuron in the medial part of the central amygdala (CeM). These neurons receive inhibitory projections from OT-receptor expressing neurons in the lateral part of the CeA (CeL)[2]. Blue-light induces endogenous release of oxytocin in the CeL that activates these GABAergic neurons producing a transient increase in GABAergic currents in the CeM neuron (upper trace) that are partially blocked by simultaneous application of the oxytocin receptor antagonist OTA (middle trace) and fully blocked by additional application of CNQX (lower trace) leaving only spontaneous inhibitory postsynaptic currents. This demonstrates *in vitro* the functional expression of our construct, similar to as we have previously shown[3]. In all graphs, the white bar represents 50 micrometers.

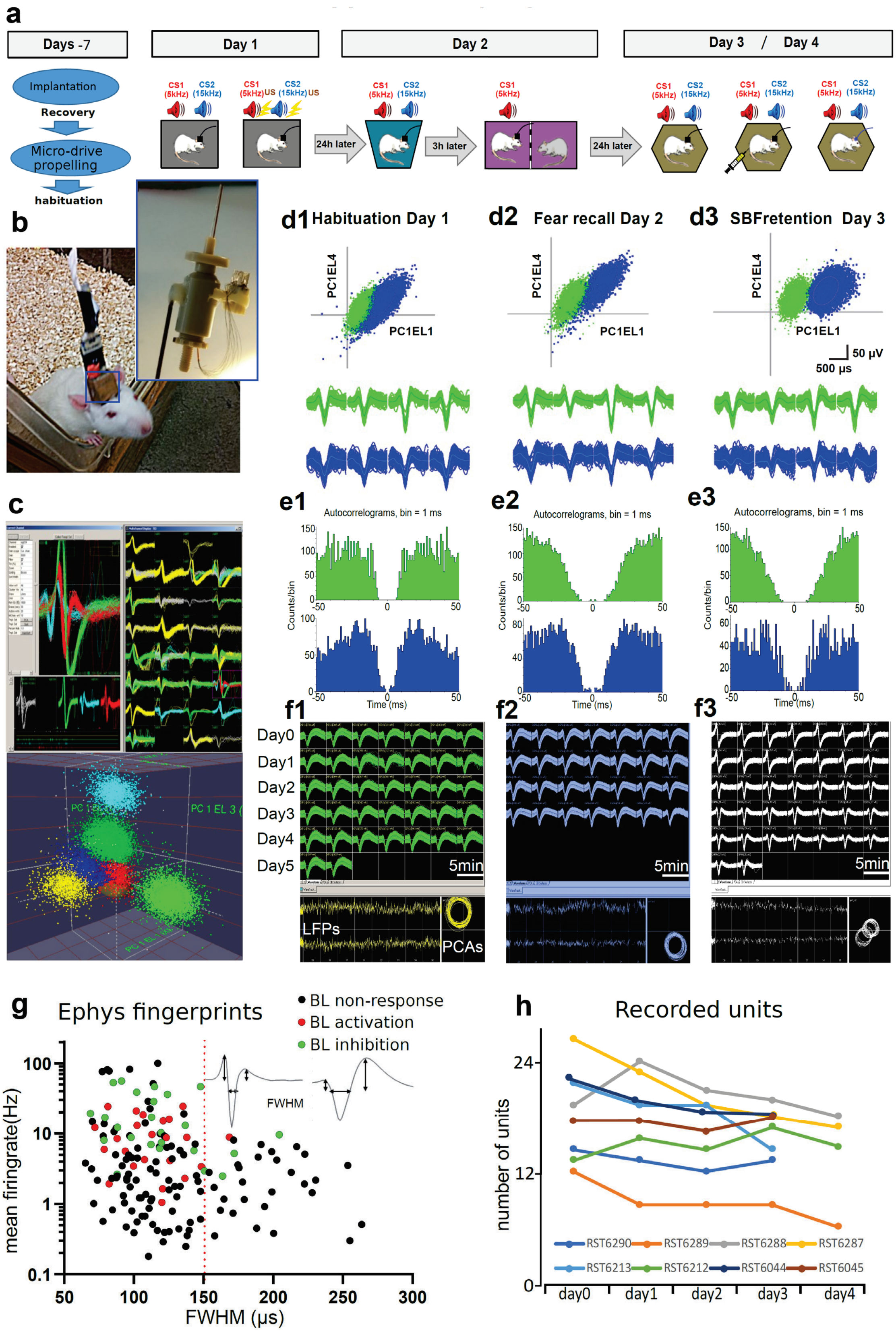

Supplementary Figure 6. *In vivo* recording protocol and data analysis pipeline demonstrates the stability across different behavioral paradigms.

(a). Standard protocol of *in vivo* optrode recording from implantation to the end of experiments (from day 0 to day3 or day4, derived from Fig 1a). **(b)** Inset: optrode with 32 channel Omnetic connectors). Metal mesh was installed around the implanted electrodes as a mini-Faraday cage to decrease electrical noise (blue square). **(c)** Upper panel: Example of online spike acquisition with Plexon system. Single unit spike waveforms were isolated by time amplitude window discrimination with a threshold 1.5 times over the baseline noise, and template matching using a multichannel acquisition processor system (RASPUTIN software, Plexon). Lower panel: We aligned the spike waveforms global minima (100 microseconds scope, maximum shift set 20) and manually chose PCA features to examine more densely clustered waveforms as a same unit (26 units maximum per rat). We visualized spatially separated waveforms as different units in principal component 3D feature projections (Fig. 6c, lower panel). **(d1-d3)** Examples of activity of two different in vivo recorded neurons over a three days behavioral protocol. (Top) 2D cluster shows two units separated through PCA analysis; the unit clusters are stable from Day 1 to Day 3. (Bottom) Color code waveforms of the two units (100 traces with their average). principal component electrode PC1EL1, PC1EL4. **(e1-e3)** Autocorrelation histogram of the two neurons in d1-d3, indicating the refractory period of units are larger than 2 ms from day1 to day3. **(f1-f3)** Examples of waveform tracking of three neurons across all data sections in 3-4 days, each column with plotted waveform showing a time bin of 10 min recording, which remains constant in f1 and f2 but gradually transformed in f3. Therefore f1-f2 were considered stable units and f3 an unstable unit that was removed from analysis. **(g)** Electrophysiological fingerprints of all the units recorded in CeA, demonstrating that most units exhibit a Full Width at Half Maximum (FWHM) of less than 150 microseconds, thereby classifying as putative interneurons[4]. (n=147 units recorded from N=8 rats). **(h)** The unit loss among 8 rats over a 5 days recording section, number of total clear sorted units dropping from 147 in the beginning to 56 in the end.

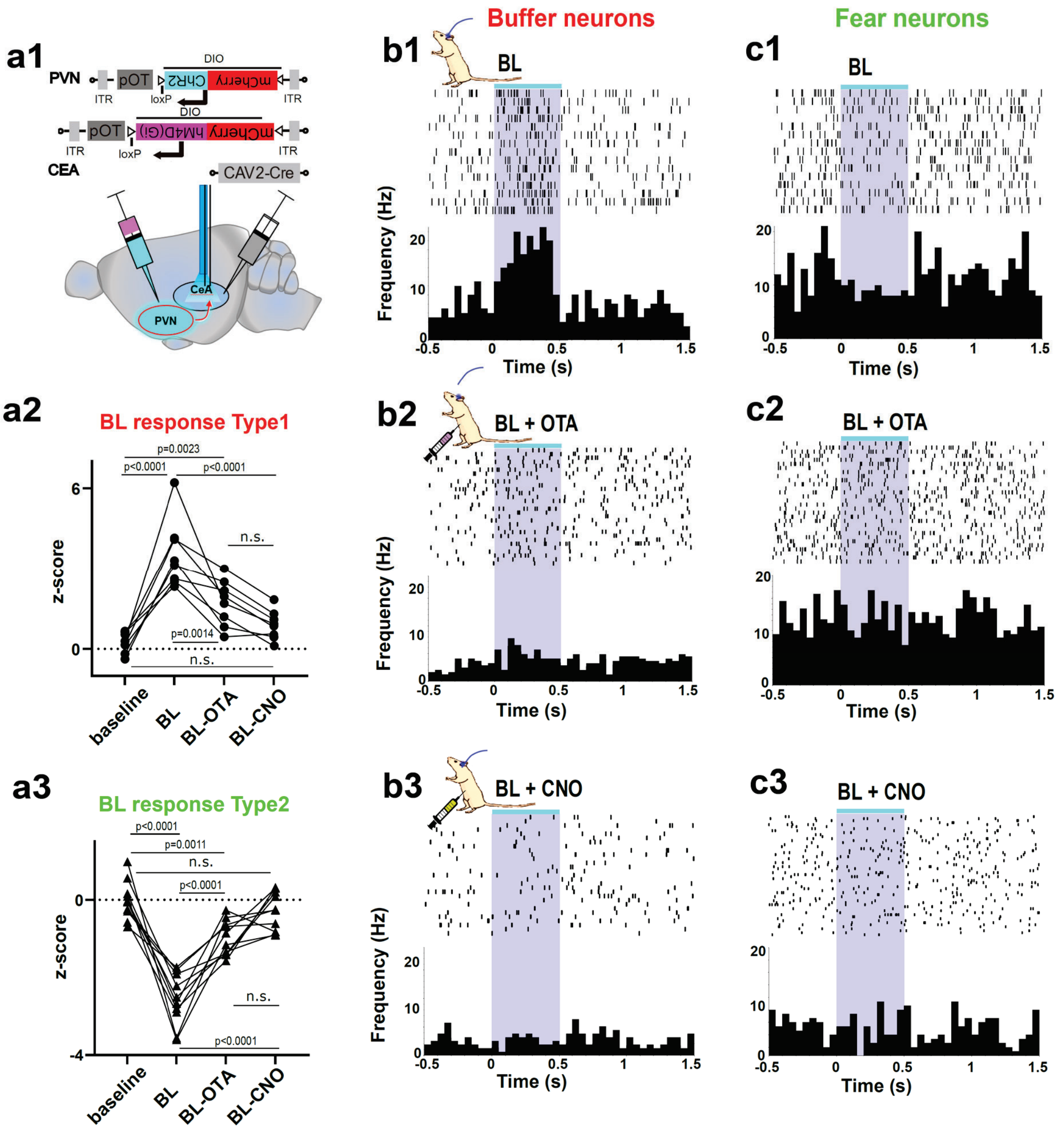

Supplementary Figure 7. Pharmacological and chemogenetic modulation of blue light responses in "buffer" and "fear" neurons in the CeA

(**a1**) Experimental set-up for virus injections to render PVN OT neurons sensitive to blue light and to CNO: double virus injection of an AAV expressing double floxed DIO mCherry-ChR2 under the OT promoter (pOT) and an AAV expressing double floxed DIO mCherry-hM4Di under the OT promoter (pOT) in the PVN, and CAV2 expressing CRE injected in the CeA. This combined genetic – retrograde labeling approach ensures ChR2 and hM4Di expression specifically in oxytocinergic neurons in the PVN that project to the CeA. (**a2**) Summary of averaged z-scores of "Buffer" (Repeated measures one-way ANOVA $F(3,21)=28.76$, $p<0.001$; Bonferroni-corrected p values in the figure) and **(a3)** "Fear" neuronal spiking

responses to blue light (BL), blue light in the presence of OTA (BL-OTA) and blue light in the presence of CNO (BL-CNO); Repeated measures one-way ANOVA $F(3,27)=42.42$, $p<0.001$, Bonferroni-corrected statistics in the figure. **(b&c)** Raster plots (top) and peri-event time histograms (bottom) BL response patterns of **(b)** a representative "Buffer" neuron and **(c)** a representative "Fear" neuron in the CeA. The raster plot represents the spikes appearing 0.5 s before and 1 s after 16 blue light exposures (at a frequency of 30 Hz during 0.5 s). **(b1)** "Buffer" neuron shows a strong excitatory response to BL, (**b2**) that is partially inhibited after IP administration of OTA and (**b3**) fully inhibited after IP administration of CNO. **(c1)** "Fear" neurons show an inhibitory response to BL **(c2)** that is partially inhibited after IP administration of OTA and **(c3)** fully inhibited after IP administration of CNO.

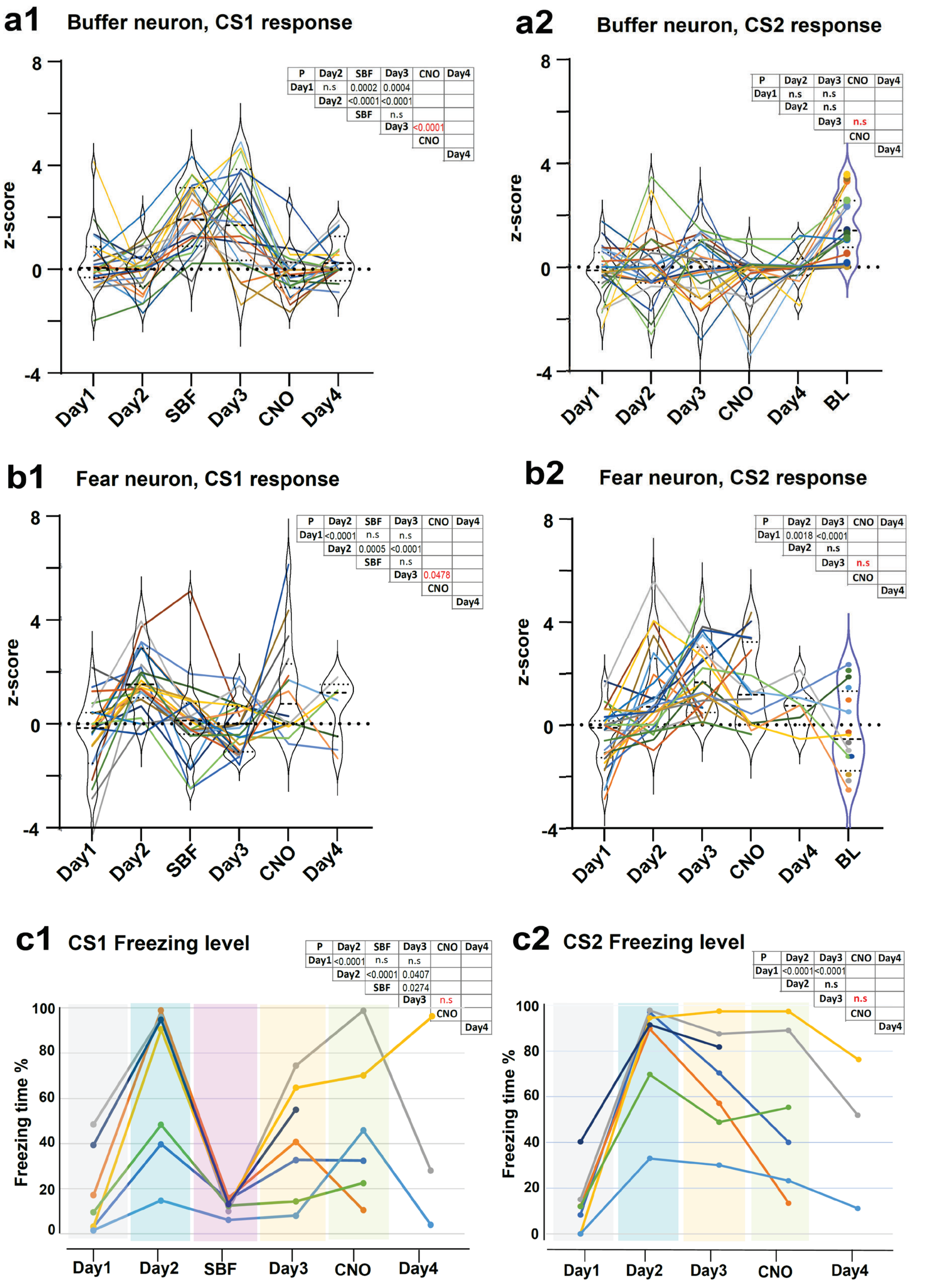

Supplementary Figure 8: Individual CS responses of Buffer and Fear neurons in the CeA and freezing levels across consecutive sessions of the social buffering paradigm

Responses of individual CeA "Buffer" neuron to CS1 and CS2 across consecutive sessions of the social buffering paradigm. Each dot and connecting line (in different colors) indicates a single neuron response, violin bars indicate the distribution of cells in each session. "Buffer" neuron responses to CS1 (**a1**) show significant changes across different sessions (D1-D3; Repeated measures one-way ANOVA F(3,69=15.85, p<0.001)), but not to CS2 (**a2,** F(2,46)=0.2; see also Figure 6). CNO in rats injected with AAV expressing hM4Di reversed the high spiking during SBF retention for CS1 but not CS2 responses. Two-sided paired Student's t-tests, t=5.387, df=19, p<0.001 for a1, and not significant for a2.**(b)** Responses of individual CeA "Fear" neurons to CS1 (**b1**) and CS2 (**b2)** across consecutive sessions of the social buffering paradigm. Fear neurons sharply increased spiking to CS1 and CS2 on Day2 followed by a steep decrease selectively to CS1 during SBF and later sections. b1, F(3,69)=13.36, p<0.001; b2, F(2,46)=17.73, p<0.001. CNO injections reversed the decreased spiking frequency to CS1, but not to CS2, observed during SBF retention. Two-sided paired Student's t-tests, t=2.156, df=15, p=0.048 for b1, and not significant for b2. **(c)** Individual freezing responses of rats from which the neurons were recorded across sessions of the SBF protocol to CS1 (**c1**) and CS2 (**c2**). F(3,18)=16.58, p<0.001 in c1, F(2,12)=45.50, p<0.001. in **c2.** There were no significant changes following injection of CNO compared with SBF retention of Day 3. Bonferroni-corrected p values are in the tables (insets), with the effects of CNO vs Day 3 (SBF retention) in red.

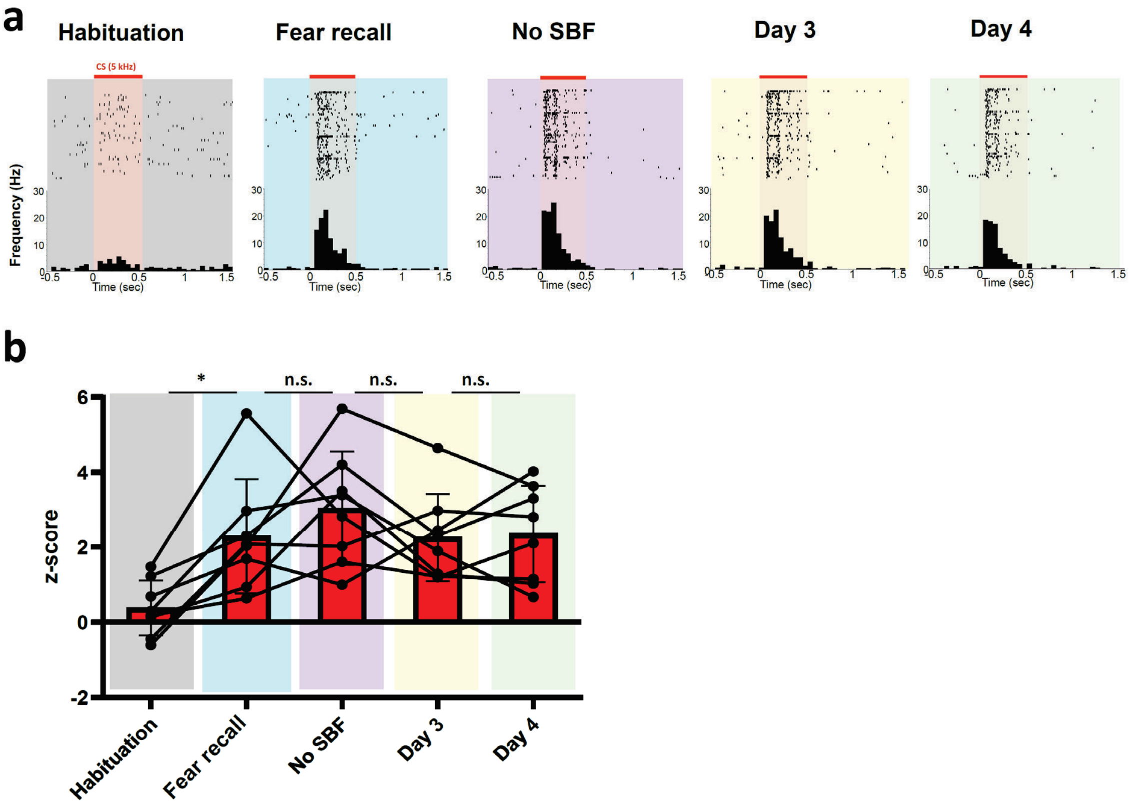

c

RM one-way ANOVA<br>Multiple comparisons									
Number of families	1								
Number of comparisons per family	10								
Alpha	0.05								
**Tukey's multiple comparisons test**	**Mean Diff.**	**95.00% CI of diff.**	**Below threshold?**	**Summary**	**Adjusted P Value**				
Habituation vs. Fear condition	-1.911	-3.501 to -0.3203	Yes	*	0.0210	A-B			
Habituation vs. No SBF	-2.663	-4.944 to -0.3816	Yes	*	0.0242	A-C			
Habituation vs. Day 2	-1.879	-4.036 to 0.2772	No	ns	0.0891	A-D			
Habituation vs. Day 3	-1.974	-3.836 to -0.1120	Yes	*	0.0384	A-E			
Fear condition vs. No SBF	-0.7525	-3.287 to 1.782	No	ns	0.8198	B-C			
Fear condition vs. Day 2	0.03111	-2.538 to 2.601	No	ns	>0.9999	B-D			
Fear condition vs. Day 3	-0.06371	-2.550 to 2.423	No	ns	>0.9999	B-E			
No SBF vs. Day 2	0.7836	-0.9227 to 2.490	No	ns	0.5179	C-D			
No SBF vs. Day 3	0.6888	-1.713 to 3.091	No	ns	0.8364	C-E			
Day 2 vs. Day 3	-0.09482	-1.346 to 1.157	No	ns	0.9985	D-E			
**Test details**	**Mean 1**	**Mean 2**	**Mean Diff.**	**SE of diff.**	**n1**	**n2**	**q**	**DF**	
Habituation vs. Fear condition	0.3708	2.281	-1.911	0.4444	8	8	6.079	7	
Habituation vs. No SBF	0.3708	3.034	-2.663	0.6376	8	8	5.907	7	
Habituation vs. Day 2	0.3708	2.250	-1.879	0.6027	8	8	4.410	7	
Habituation vs. Day 3	0.3708	2.345	-1.974	0.5205	8	8	5.364	7	
Fear condition vs. No SBF	2.281	3.034	-0.7525	0.7084	8	8	1.502	7	
Fear condition vs. Day 2	2.281	2.250	0.03111	0.7181	8	8	0.06126	7	
Fear condition vs. Day 3	2.281	2.345	-0.06371	0.6950	8	8	0.1296	7	
No SBF vs. Day 2	3.034	2.250	0.7836	0.4769	8	8	2.324	7	
No SBF vs. Day 3	3.034	2.345	0.6888	0.6714	8	8	1.451	7	
Day 2 vs. Day 3	2.250	2.345	-0.09482	0.3498	8	8	0.3834	7	

Supplementary Fig 9. Fear conditioning without SBF protocol shows no signs of single unit extinction in 3 consecutive days.

We recorded single unit responses in 3 rats that had undergone fear conditioning and "Fear recall" without subsequent SBF exposure which was replaced by only 4 times CS1 tones exposures ("No SBF"). The CeA "Fear neurons" were identified by their increased single unit firing responses after fear conditioning on Day 2 ("Fear recall"). a). Raster (top) and frequency (bottom) plot example of single unit firing response to CS (5 kHz) (0.5sec, 5Hz, red shade) across different sessions as in Figure 6. b. Individual and average z-scores of 8

neurons, recorded in 3 rats showing, upon extra exposure to the CS without SBF ("no SBF"), no significant decrease signs of extinguished responses to the CS the next days "Day 3" nor on "Day 4" consistent with maintained responses to the CS without signs of extinction. Significance was only detected between "habituation" and "fear recall" (after fear conditioning. (*p<0.05, n=8 cells, background colors as in Fig. 6). c) repeated measures one way ANOVA, Bonferroni corrections for multiple comparisons, of data in b.

Brain Regions targeted by the PVN	
**Olfactory System**	Anterior olfactory nucleus (medial & ventral); Olfactory tubercle; Island of Calleja; Lateral entorhinal cortex
**Cortical areas**	Nucleus of the horizontal limb of the diagonal band; Frontal association cortex; dorsal peduncular cortex; dorsal/ventral taenia tecta; medial and ventral orbital cortex; prelimbic, cingulate and insular cortices; frontal and temporal association cortices
**Basal ganglia**	Nucleus accumbens shell and core; Caudate putamen; Globus pallidus
**Limbic system**	Lateral septum, bed nucleus of the stria terminalis; CA1, CA3 and dentate gyrus of hippocampus; subiculum; medial, central and basolateral amygdala
**Thalamus**	Paraventricular thalamic nucleus
**Hypothalamus**	Supraoptic nucleus
**Pituitary**	Posterior lobe

Table 1 | Brain regions targeted by OTergic projections from the PVN. OTergic neurons project throughout the brain, including to regions involved in social behavior and fear. By targeting these projections by optogenetic and chemogenetic means we found that modulation of the OT system across the brain had similar effects on freezing in our behavioral paradigm as modulation of OT signaling in the CeA directly (compare Fig. 1b with Fig. 3). This suggests that OT signaling in the CeA is key to SBF, although contributions of other brain regions cannot be excluded. The table is based on our previous characterization of OT-positive projections from the PVN[3].

Virus	Volume Injected	Titer	Origin
rAAV-pOT-hM4D(Gi)-mCherry (Serotype 1/2)	500nl	~1x10e10 vg/ml	Donation V. Grinevich (catalog :A181)
rAAV-pOT-ChR2-mCherry (Serotype 1/2)	500nl	~1x10e10 vg/ml	Donation V. Grinevich (catalog :A89)
rAAV-pOT-DIO-hM4D(Gi)-mCherry (Serotype 1/2)	300nl	~1x10e10 vg/ml	Donation V. Grinevich (catalog :A261)
rAAV-pOT-DIO-ChR2-mCherry (Serotype 1/2)	300nl	3,86x10e10 vg/ml	Donation V. Grinevich (catalog :A188)
CAV2-CRE (Serotype 2)	500nl	Delivery at 6.9x10 e12 vg/ml Diluted to 2~3 x10e12 vg/ml	Montpellier vectorology platform(PVM), Biocampus Montpellier,. France

* Virus Genome copies per ml vg/ml, which also written in physical particles pp
* each time PVM may deliver different virus titres, which need different dilution ratio. E.g. last time we ordered 2.85 dose of 2.5x10e12 pp about 50ul, and PVM delivered 40ul of 6.9x10e12 vg/ml

Table 2 | Information on the viruses used in the present study

References

1. Grund, T. *et al.* Chemogenetic activation of oxytocin neurons: Temporal dynamics, hormonal release, and behavioral consequences. *Psychoneuroendocrinology* **106**, 77–84 (2019).
2. Huber, D., Veinante, P. & R. Stoop. Vasopressin and oxytocin excite distinct neuronal populations in the central amygdala. *Science* **308**, 245–8 (2005).
3. Knobloch, H. S. *et al.* Evoked axonal oxytocin release in the central amygdala attenuates fear response. *Neuron* **73**, 553–566 (2012).
4. Blot, A. *et al.* Time-invariant feed-forward inhibition of Purkinje cells in the cerebellar cortex *in vivo*: Interneurons *in vivo*. *J Physiol* **594**, 2729–2749 (2016).
